# Supplementary material for: Development of a longevous two-species biophotovoltaics with constrained electron flow
Source: Nat Commun. 2019 Sep 19;10:4282. doi: 10.1038/s41467-019-12190-w (PMC6753107; doi:10.1038/s41467-019-12190-w)
Supplement: Supplementary file 1 — Supplementary Information [file 41467_2019_12190_MOESM1_ESM.pdf]

Development of a longevous two-species biophotovoltaics with constrained electron flow

Zhu *et al*.

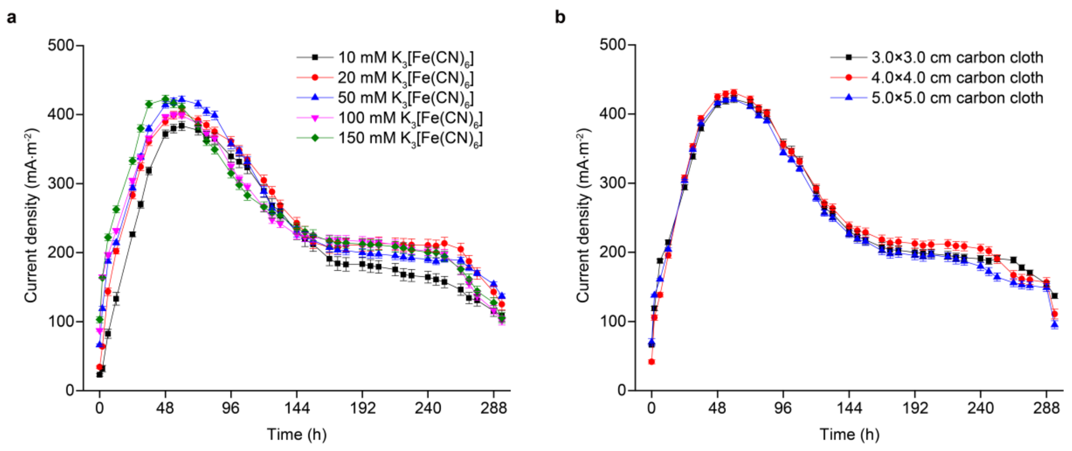

Supplementary Fig. 1. Cathodic limitation on current production in the setups of this study. **a**, Current density produced by strain *S. oneidensis*-WT using different concentrations of ferricyanide in 50 mM potassium phosphate buffer as cathodic electrolyte. **b**, Current density produced by strain *S. oneidensis*-WT using different sizes of carbon cloth as cathode. 15 mM sodium DL-lactate was used as electron donor for all setups. Error bars represent the standard deviations from n = 3 independent experiments. Source data are provided as a Source Data file.

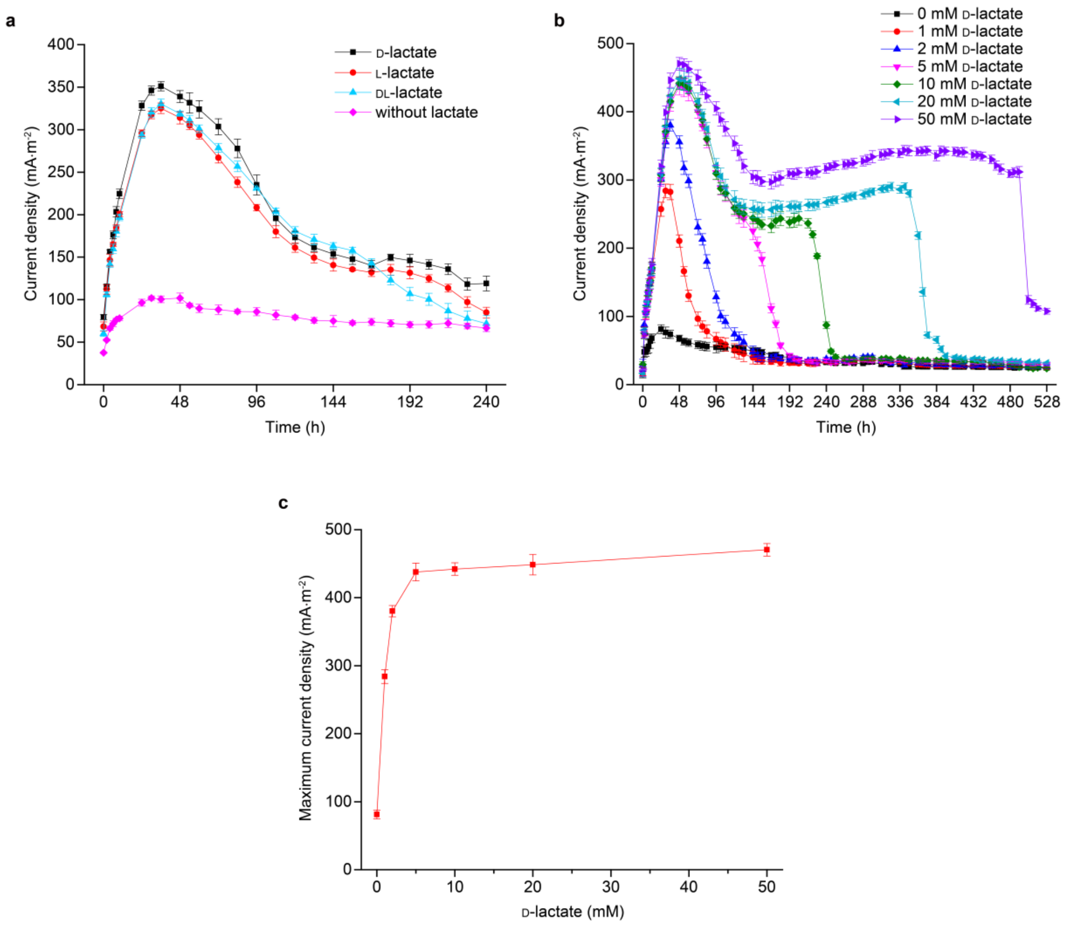

Supplementary Fig. 2. Current production in mono-culture of strain MR-1. **a**, Current density produced by strain *S. oneidensis*-WT in M9 medium supplemented with 15 mM sodium D-lactate, 15 mM sodium L-lactate, 15 mM sodium DL-lactate or without lactate as electron donor, respectively. **b**, Current density produced by strain *S. oneidensis*-WT in M9 medium supplemented with different concentrations of sodium D-lactate as electron donor. **c**, The maximum current density versus the concentration of D-lactate, derived from (**b**). Error bars represent the standard deviations from n = 3 independent experiments. Source data are provided as a Source Data file.

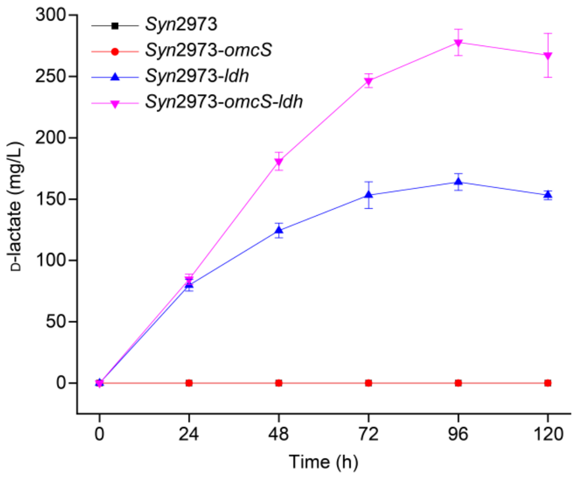

Supplementary Fig. 3. D-lactate production of the engineered cyanobacteria in BG11 medium. The genetically engineered cyanobacteria were cultured in 50 mL BG11 medium in a 100-mL flask at 38°C at 140 rpm. The concentration of D-lactate was quantified by HPLC every 24 h. Error bars represent the standard deviations from n = 3 independent experiments. Source data are provided as a Source Data file.

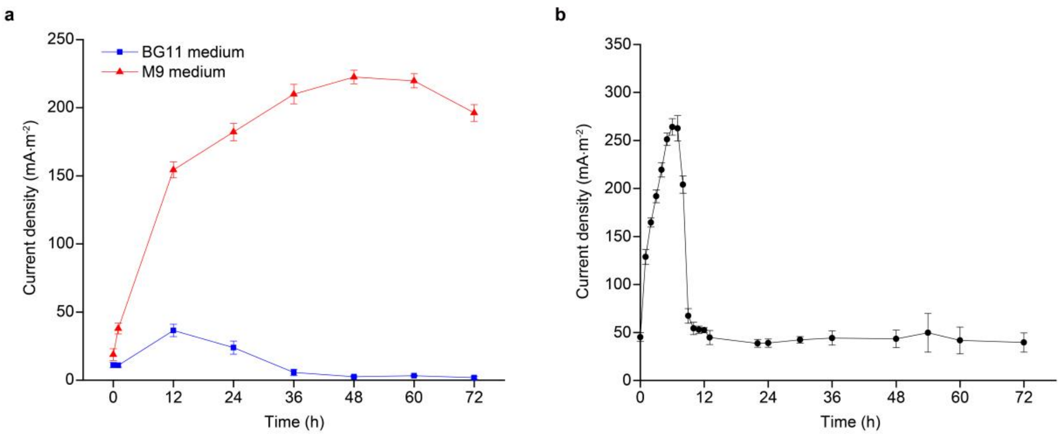

Supplementary Fig. 4. Current production by mono-culture and co-culture in different environments. a, Current density produced by mono-culture of strain *S. oneidensis*-WT in BG11 and M9 media. 15 mM sodium D-lactate was used as electron donor. **b**, Current density produced by direct co-culture of two species under light. The strain *Syn*2973-*omcS*-*ldh* was cultured in anodic chamber for 2 days with a stirring at 250 rpm, and then the strain *S. oneidensis*-Δ*napA* was inoculated into the anodic chamber for current production without stirring. The anodic chamber was incubated under light during the whole process. Error bars represent the standard deviations from n = 3 independent experiments. Source data are provided as a Source Data file.

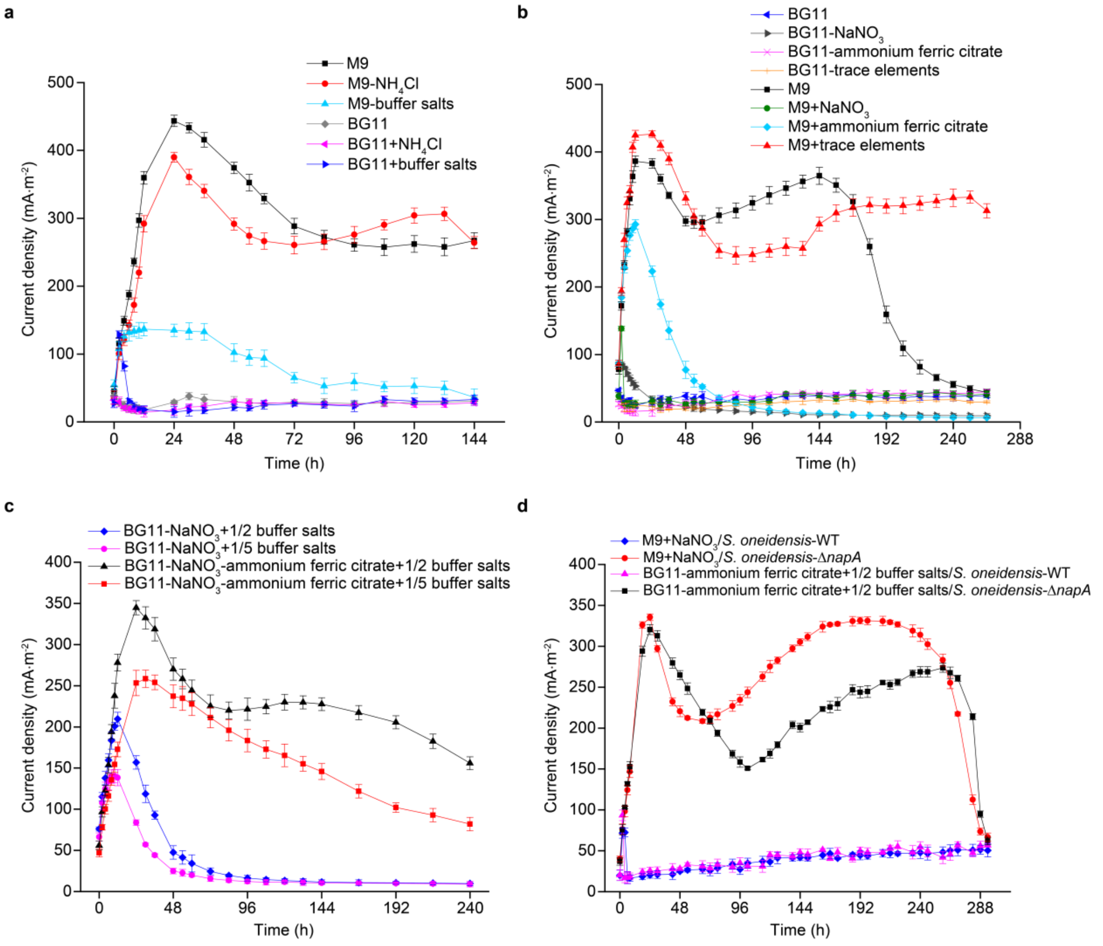

Supplementary Fig. 5. Modifying BG11 medium to support current production of *S. oneidensis*. **a**, The effects of ammonium chloride and buffer salts on the current production of strain *S. oneidensis*-WT. **b**, The effects of sodium nitrate, ammonium ferric citrate and trace elements on the current production of strain *S. oneidensis*-WT. **c**, Further verification on the effects of sodium nitrate, ammonium ferric citrate and buffer salts on the current production of strain *S. oneidensis*-WT. **d**, Current production by strain *S. oneidensis*-WT and strain *S. oneidensis*-Δ*napA* in the modified media. All setups were supplemented with 15 mM sodium D-lactate as electron donor. Error bars represent the standard deviations from n = 3 independent experiments. Source data are provided as a Source Data file.

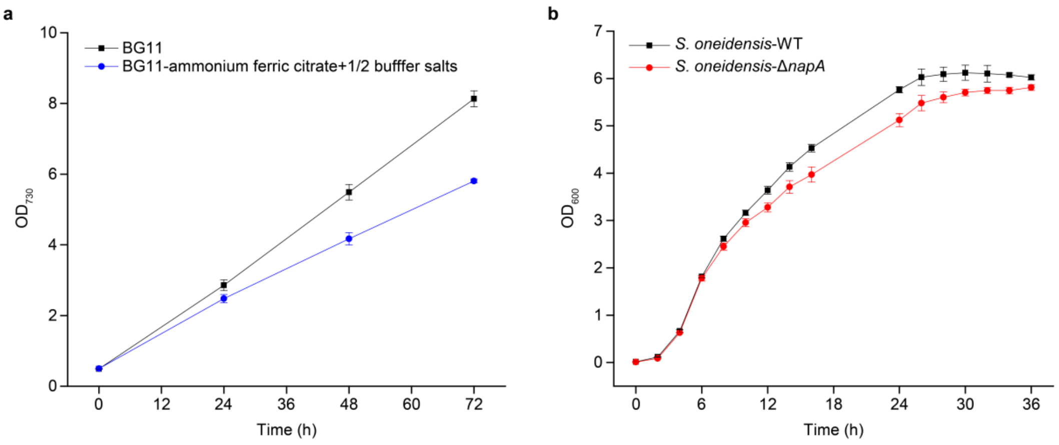

Supplementary Fig. 6. The growth of cyanobacteria and *S. oneidensis*. a, The growth of strain *Syn*2973-*omcS-ldh* in BG11 medium and a modified BG11 medium (without ammonium ferric citrate, but with addition of 1/2 buffer salts of that in M9). The strain was cultured in 50 mL medium in a 100-mL flask at 38°C at 140 rpm. The cell density at OD_{730} was measured every 24 h. **b**, Growth comparison of strain *S. oneidensis*-WT and strain *S. oneidensis*-Δ*napA*. Two strains were cultured in 40 mL LB medium in a 100-mL flask at 30°C at 200 rpm. The cell density at OD_{600} was measured. Error bars represent the standard deviations from n = 3 independent experiments. Source data are provided as a Source Data file.

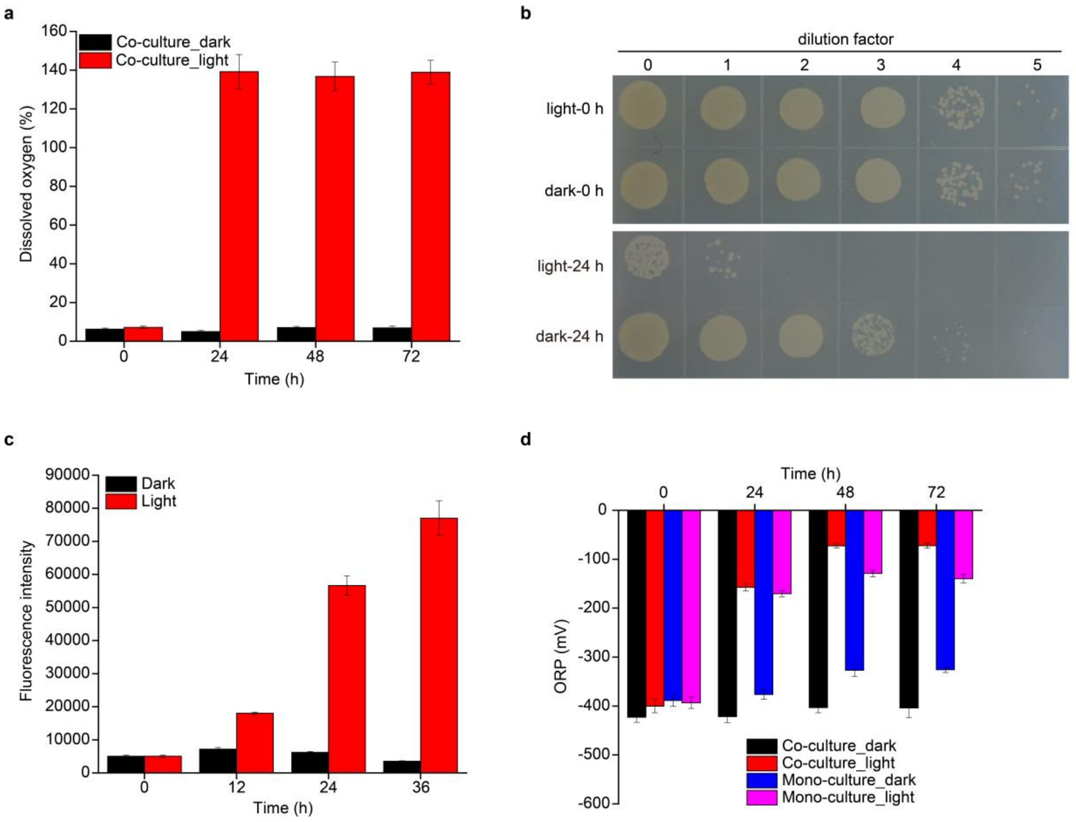

Supplementary Fig. 7. The effect of illumination on the mono-cultures and co-cultures. a, The relative dissolved oxygen (DO) levels of the co-cultures under light and dark conditions. The oxygen concentration in the air was set as 100%. **b**, The plate drop test revealed a growth defect of strain *S. oneidensis*-Δ*napA* under light condition. The strain *S. oneidensis*-Δ*napA* was cultured in MBG11 medium at 30°C under either light or dark conditions with an initial OD_{600} of 0.25, which was set as the undiluted culture (dilution factor 0), and 10-fold serial dilutions were prepared with sterile water. Five microliters of each dilution was dropped onto LB plates, which were incubated under dark at 30°C for colony formation. **c**, Intracellular reactive oxygen species (ROS) levels of strain *S. oneidensis*-Δ*napA* under light and dark conditions. The ROS level is positively correlated with the fluorescence intensity[1]. **d**, The oxidation-reduction potential (ORP) levels of mono-cultures and co-cultures under light and dark conditions. ORP value was measured using a handheld ORP Meter by immersing the probe into the cell culture and the result was displayed directly. Error bars represent the standard deviations from n = 3 independent experiments. Source data are provided as a Source Data file.

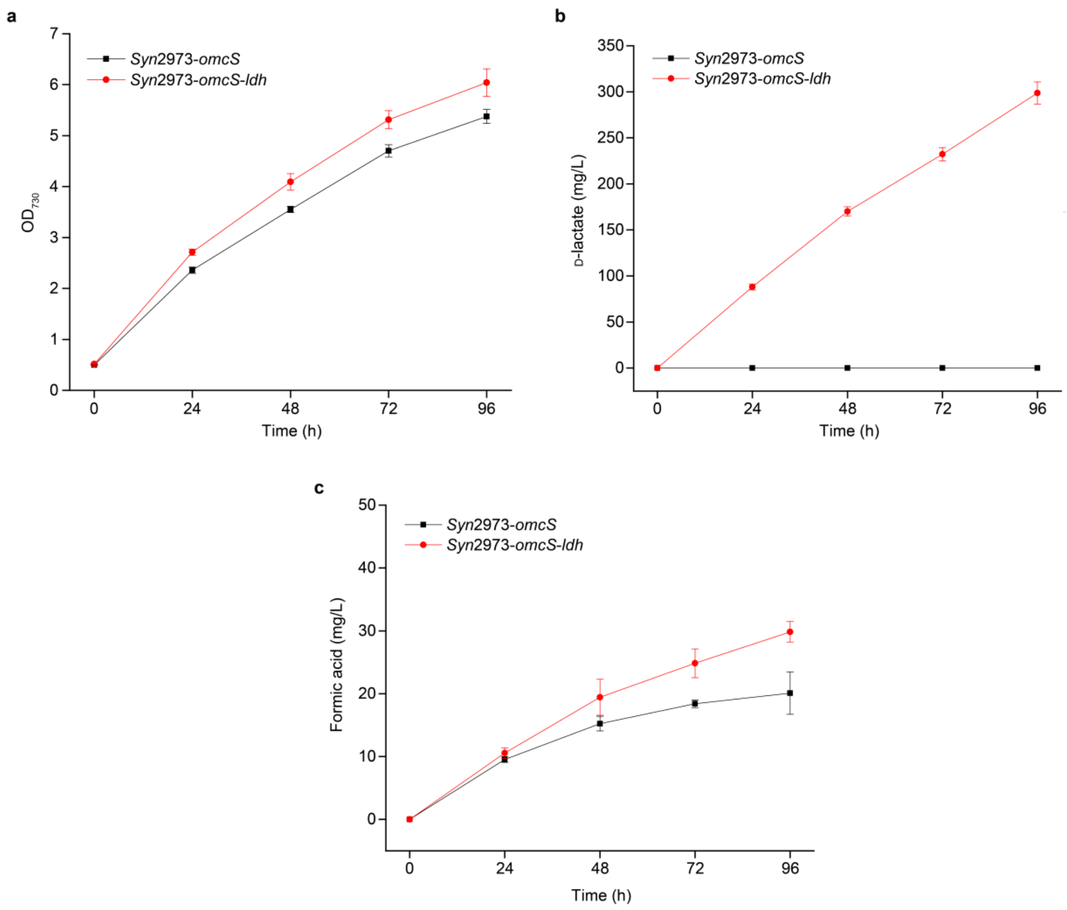

Supplementary Fig. 8. Growth and D-lactate production of the engineered cyanobacteria in MBG11 medium. The strains *Syn*2973-*omcS* and *Syn*2973-*omcS-ldh* were cultured in 50 mL MBG11 medium in a 100-mL flask at 38°C at 140 rpm. **a**, The cell density at OD_{730} was measured every 24 h. **b**, D-lactate titer was measured every 24 h. **c**, Formic acid titer was measured every 24 h. Error bars represent the standard deviations from n = 3 independent experiments. Source data are provided as a Source Data file.

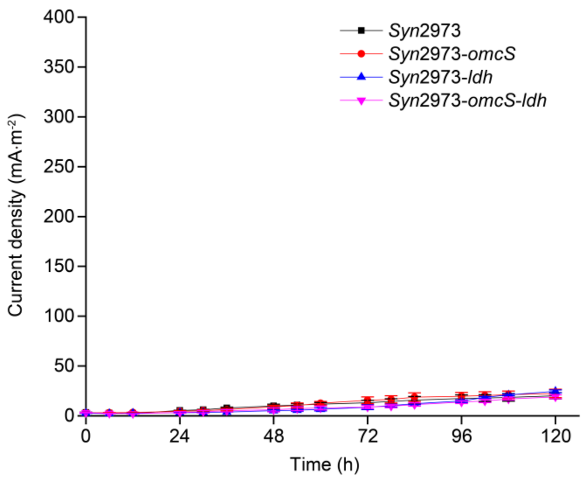

Supplementary Fig. 9. Current production by mono-cultures of the engineered cyanobacteria in dual-chamber devices. The cyanobacterial cultures after cultivation in MBG11 medium for 48 h were used as the anodic electrolytes, and the cell density was adjust to OD_{730} = 3.5. Error bars represent the standard deviations from n = 3 independent experiments. Source data are provided as a Source Data file.

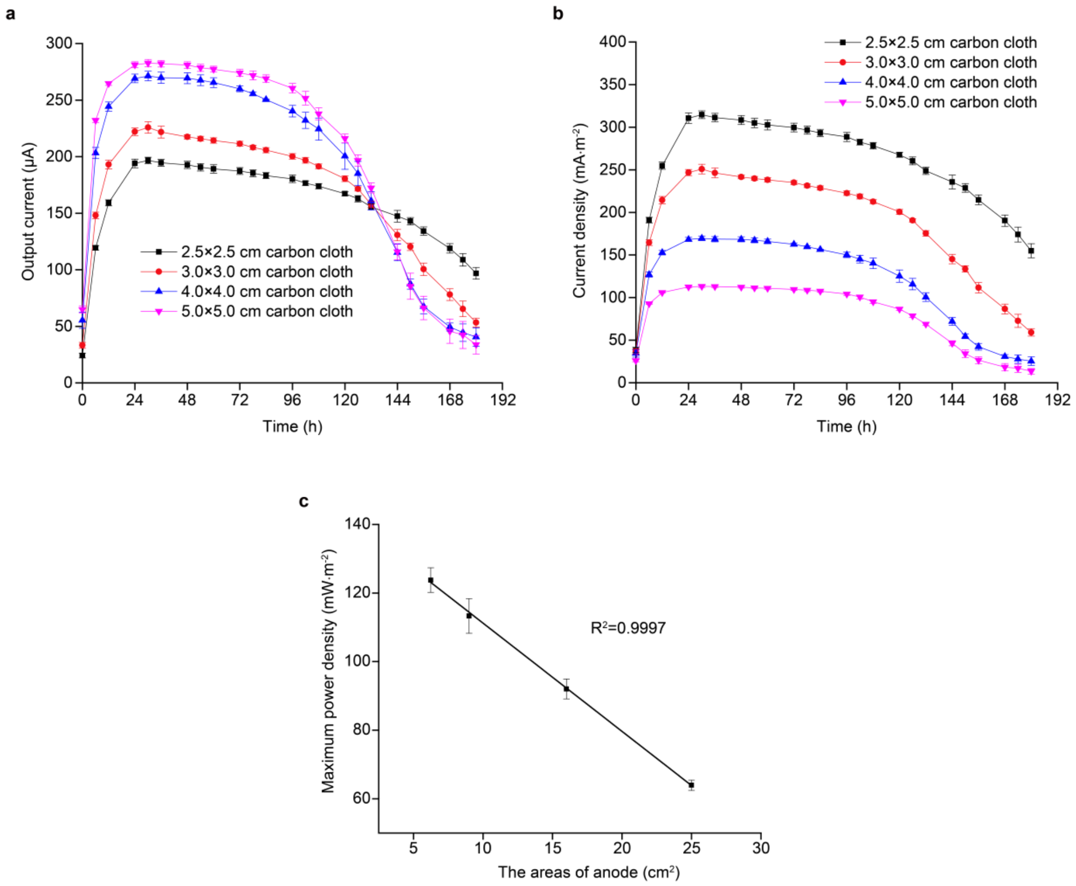

Supplementary Fig. 10. Current production by the CLS microbial consortium in the setups with different sizes of anode. a, Current output in the setups with different sizes of anode. **b**, Current density in the setups with different sizes of anode. **c**, The maximum power density versus the geometric area of anode. The CLS microbial consortium was constructed with temporal separation organization. Error bars represent the standard deviations from n = 3 independent experiments. Source data are provided as a Source Data file.

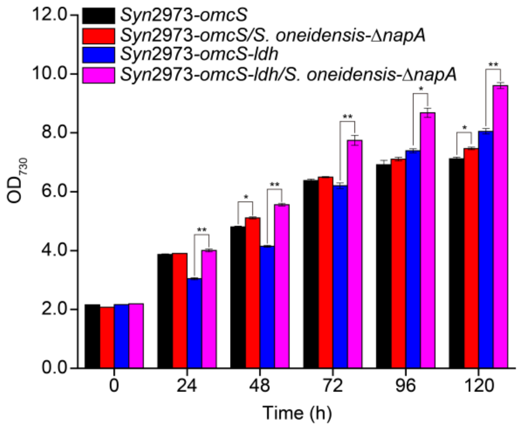

Supplementary Fig. 11. Defined microbial consortium facilitated the growth of cyanobacteria. The experiment was conducted in spatial separation setups. The cell density of cyanobacteria in axenic cultures (cyanobacteria only) and co-cultures was measured at appointed time points. Axenic cultures include the mono-culture of *Syn*2973-*omcS* and the mono-culture of *Syn*2973-*omcS-ldh*. Co-cultures include the CS microbial consortium (*Syn*2973-*omcS*/*S. oneidensis*-Δ*napA*) and the CLS microbial consortium (*Syn*2973-*omcS-ldh*/*S. oneidensis*-Δ*napA*). Error bars represent the standard deviations from n = 2 independent experiments. *P*-values of two-sample *t*-tests are denoted with asterisks: * <0.05, ** <0.01. Source data are provided as a Source Data file.

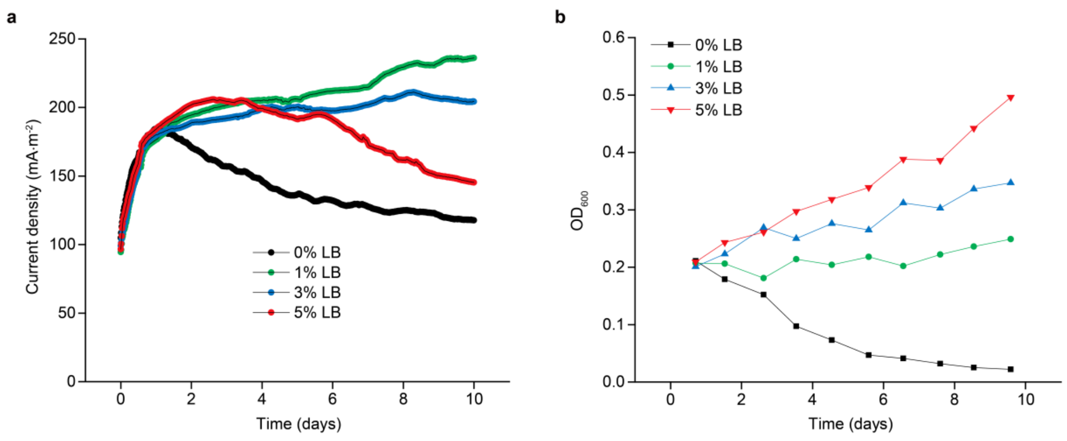

Supplementary Fig. 12. Current production in mono-culture of strain *S. oneidensis*-Δ*napA* with medium replenishment. The MBG11 media containing 3.5 mM D-lactate with different LB additions (0%, 1%, 3% and 5%) were used to replenish the anodic electrolytes. **a**, Current density produced in the replenished setups. Current density was recorded every 20 minutes. **b**, The cell density of *S. oneidensis*-Δ*napA* in the anodic chambers during the whole process. Source data are provided as a Source Data file.

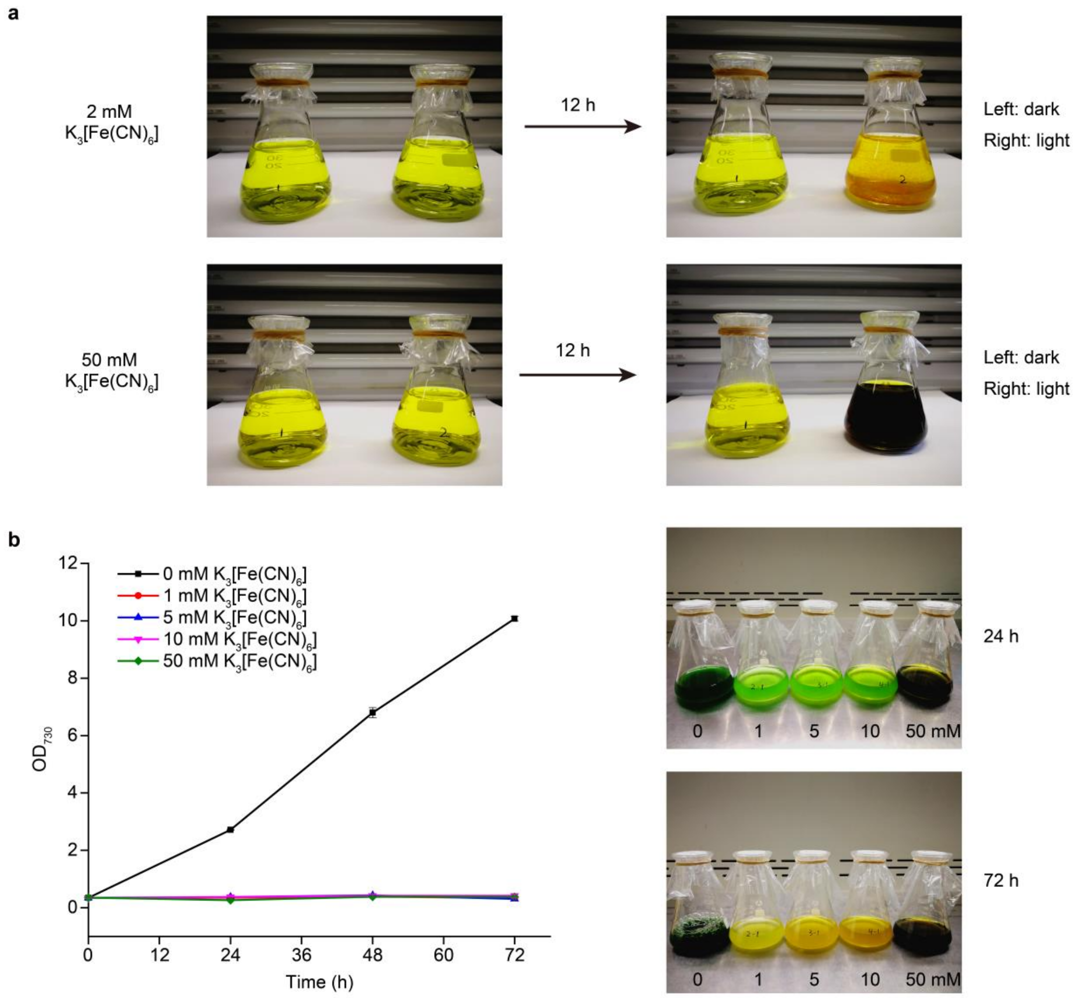

Supplementary Fig. 13. Decomposition of potassium ferricyanide under illumination and the toxicity of its decomposed products on the growth of cyanobacteria. a, Potassium ferricyanide (aqueous solutions with concentration of 2 mM and 50 mM) were decomposed to $Fe(OH)_3$ and KCN after exposure to white light for 12 h according to the previous study[2]. **b**, The decomposed products of ferricyanide inhibited the growth of cyanobacteria and finally killed the cells even at a low concentration of 1 mM under illumination condition. The source data of Supplementary Fig. 13b are provided as a Source Data file.

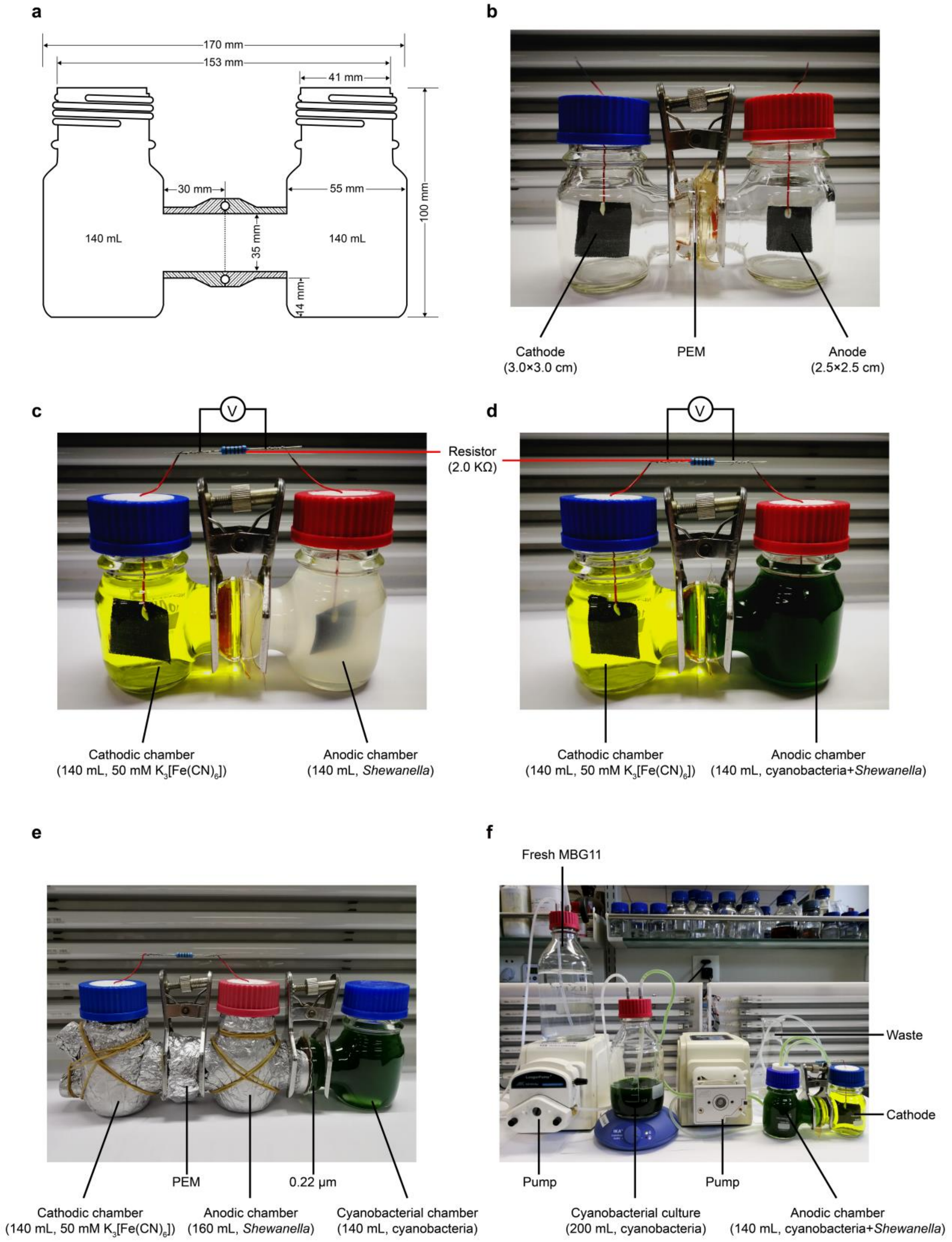

Supplementary Fig. 14. Diagram and photographs of the experimental setups used in this study. a, The diagram of dual-chamber electrochemical device, showing the actual geometry and size. **b**, A dual-chamber electrochemical device (empty) shows the sizes of anode and cathode. **c**, The dual-chamber device used for constructing the mono-culture of *S. oneidensis*. The anode and cathode are connected using a 2.0 kΩ external resistor, and the voltage (*U*) across the resistor can be

measured. **d**, The dual-chamber device used for constructing temporally separated microbial consortium. Cyanobacteria and *S. oneidensis* stay together in the anode chamber during the discharging stage. **e**, The three-chamber device used for constructing the spatially separated microbial consortium. The anodic and cathodic chambers are covered with the tin foil to shield them from light. The cyanobacterial chamber and anodic chamber are separated by a micro-porous membrane with 0.22-μm pores. **f**, The spatial-temporal separation setup with medium replenishment. The fresh MBG11 medium is continually pumped into the cyanobacterial culture, which is subsequently pumped out and flow into the anodic chamber for current production. PEM: proton exchange membrane.

Supplementary Table 1. Composition of the media used in this study.

Medium components (g/L)	M9	BG11	MBG11
$Na_2HPO_4 \cdot 12H_2O$	17.1		3.42
KH_2PO_4	3.0		0.6
NaCl	0.5		
NH_4Cl	1.0		
$MgSO_4$	0.24	0.036	0.036
$CaCl_2$	0.011	0.027	0.027
$NaNO_3$		1.5	1.5
$K_2HPO_4 \cdot 3H_2O$		0.047	0.047
Na_2CO_3		0.02	0.02
Citrate		0.006	0.006
Ammonium ferric citrate		0.006	
EDTA disodium		0.001	0.001
*Trace elements solution (1000×)		1.0 mL	1.0 mL

*(per liter): 2.86 g H_3BO_3, 1.81 g $MnCl_2 \cdot 4H_2O$, 0.222g $ZnSO_4 \cdot 7H_2O$, 0.079 g $CuSO_4 \cdot 5H_2O$, 0.391 g $Na_2MoO_4 \cdot 2H_2O$ and 0.04 g $CoCl_2 \cdot 6H_2O$.

Supplementary References

1. Wang, H. & Joseph, J. A. Quantifying cellular oxidative stress by dichlorofluorescein assay using microplate reader. *Free Radic. Biol. Med.* **27,** 612-616 (1999).
2. Foster, G. W. A. The action of light on potassium ferrocyanide. *J. Chem. Soc.* **89,** 912-920 (1906).
