# Supplementary material for: Development of a longevous two-species biophotovoltaics with constrained electron flow
Source: Nat Commun. 2019 Sep 19;10:4282. doi: 10.1038/s41467-019-12190-w (PMC6753107; doi:10.1038/s41467-019-12190-w)
Supplement: Supplementary file 4 — Description of Additional Supplementary Files [file 41467_2019_12190_MOESM4_ESM.pdf]

Description of Additional Supplementary File

File Name: Supplementary Data 1

Description: The summary of previously reported BPV devices (by using living microorganisms) on their basic information and performances.
